# SIK1 inhibits IL-1β-stimulated cartilage apoptosis and inflammation *in vitro* through the CRTC2/CREB1 signaling

**DOI:** 10.1515/biol-2022-1016

**Published:** 2025-03-06

**Authors:** Mangmang Chen, Luyou Ye, Shenglei Lin

**Affiliations:** Department of Orthopedics Surgery, The Dingli Clinical Institute of Wenzhou Medical University, Wenzhou Central Hospital, No. 252 Baili East Road, Lucheng, Wenzhou, Zhejiang, 325000, China

**Keywords:** osteoarthritis, SIK1, chondrocyte apoptosis, inflammation, CRTC2/CREB1 signaling

## Abstract

Osteoarthritis (OA) is a chronic degenerative joint disease that affects 70–90% of individuals over the age of 75 and over 100 million people globally. Current treatments primarily offer symptomatic relief and do not effectively halt disease progression, highlighting the need for improved therapeutic strategies. Salt-inducible kinase 1 (SIK1) plays a role in regulating key physiological processes, including gluconeogenesis, glycolysis, and bone metabolism. Despite these insights, the specific role and underlying mechanisms of SIK1 in OA pathogenesis remain inadequately understood. This study aims to elucidate the function of SIK1 in OA cells. We observed that SIK1 was downregulated in a cell model of OA. The overexpression of SIK1 was found to inhibit IL-1β-induced chondrocyte apoptosis and inflammation. Additionally, SIK1 overexpression enhanced the activation of the CRTC2/CREB1 axis, suggesting a protective role for SIK1 in cartilage cells. In summary, SIK1 exerts a protective effect against IL-1β-induced cartilage apoptosis and inflammation *in vitro* through the CRTC2/CREB1 signaling axis.

## 1 Introduction

Osteoarthritis (OA) is a chronic degenerative joint disease that significantly contributes to disability among the elderly [1]. The prevalence of OA is alarmingly high, affecting approximately 70–90% of individuals over the age of 75, with more than 100 million people globally suffering from this condition [2]. Clinically, OA is characterized by joint pain, swelling, deformity, and limited mobility [3]. The primary pathological features of OA include cartilage degradation, destruction, and the formation of osteophytes [3]. Despite its widespread impact, current pharmacological treatments, such as analgesics, primarily offer symptomatic relief and do not effectively prevent or slow disease progression [2,4]. The development of OA is closely associated with cartilage tissue lesions, and chondrocyte apoptosis is a key factor in cartilage damage. The disease presents a multifactorial etiology and complex pathogenesis [4], highlighting the urgent need for the development of more effective targeted therapies.

Research has established a strong positive correlation between chondrocyte apoptosis and the severity of OA. Reactive oxygen species (ROS) are crucial mediators of chondrocyte apoptosis [5]. It is known that excessive ROS accumulation can lead to mitochondrial dysfunction, which in turn promotes apoptosis in cartilage cells [6]. Recent studies have increasingly highlighted the central role of ROS in the pathogenesis of OA, particularly through the mechanisms of chondrocyte apoptosis and extracellular matrix degradation. This degradation involves both reduced matrix synthesis and increased matrix decomposition [6]. Additionally, inflammation-related osteoporosis, characterized by bone mass loss and damage to bone microstructure due to chronic infections or autoimmune diseases, further complicates the disease. Therefore, strategies aimed at inhibiting apoptosis and reducing ROS levels may provide new therapeutic avenues for the treatment of OA.

Salt-inducible kinase 1 (SIK1) is a serine/threonine kinase protein expressed across multiple tissues [7]. It belongs to the adenosine monophosphate-activated protein kinase family of serine/threonine kinases. It has been reported that inhibiting SIK1 could be a novel therapeutic approach for modulating pro-inflammatory and immunomodulatory pathways, with potential applications in treating inflammatory diseases [7–9]. SIK1 is essential for regulating various cellular processes, including electrolyte balance, carbohydrate and lipid metabolism, cell proliferation, and circadian rhythms [8–10]. For example, SIK1 regulates CRTC2-mediated gluconeogenesis under both physiological and high glucose conditions [11]. Targeting the SIK1-CRTC2 axis presents a potential strategy for managing diabetes [12]. Additionally, SIK1 activation by phanginin A has been shown to inhibit gluconeogenesis by enhancing PDE4 function and blocking the cAMP/PKA/CREB axis [13]. Furthermore, SIK1 and its isoform SIK3 influence aerobic glycolysis and breast cancer cell growth by targeting the p53 and mTOR pathways [14]. In the context of bone metabolism, SIK1 acts as a critical negative regulator of pre-osteoblast proliferation and osteoblast differentiation, with its inhibition being essential for BMP2 signaling during osteogenesis [10]. Recent studies suggest that SIK1 may also be a novel target for OA [15]. Despite these insights, the specific role and underlying mechanisms of SIK1 in OA remain poorly understood.

In this study, we aim to investigate the function of SIK1 in an *in vitro* model of OA, specifically focusing on its potential role in modulating chondrocyte apoptosis and inflammation. We hypothesize that SIK1 inhibits IL-1β-induced cartilage apoptosis and inflammation *in vitro*. Elucidating the role of SIK1 could lead to the development of novel therapeutic strategies for managing OA, potentially improving outcomes for patients affected by this debilitating condition.

## 2 Materials and methods

### 2.1 Cell culture and treatment

The human chondrocyte cell line C28/I2 (HTX2308) was purchased from ATCC and cultured in DMEM/F-12 medium (Gibco, USA) supplemented with 10% FBS (Gibco, USA). The cells were maintained at 37°C in a 5% CO_2_ atmosphere. For the OA model, cells were treated with IL-1β (10 ng/mL, PeproTech, USA) for 24 h.

### 2.2 Viral infection

C28/I2 cells were infected with either ad-SIK1 or ad-NC, both purchased from GeneChem (Shanghai, China). Cell infection was conducted at a multiplicity of infection of 50 in the presence of 5 µg/mL polybrene (Beyotime, ST1382). After 24 h, the medium was replaced with fresh growth medium.

### 2.3 Western blot analysis

The proteins were separated by SDS-PAGE and transferred onto PVDF membranes (Millipore, USA). The membranes were blocked with 5% non-fat milk and incubated overnight at 4°C with primary antibodies. The primary antibodies used were SIK1 (1:1,000; ab62738, Abcam, UK), CRTC2 (1:1,000, ab236134; Abcam, UK), p-CRTC2 (1:1,000, ab76477; Abcam, UK), CREB1 (1:1,000, 9197; Cell Signaling Technology, USA), p-CREB1 (1:1,000, 9198; Cell Signaling Technology, USA), and β-actin (1:5,000, AF7018; Affinity Biosciences, USA). After washing, membranes were incubated with HRP-conjugated secondary antibodies (1:5,000; Beyotime, China) for 1 h, and the bands were visualized using an ECL detection kit (Beyotime, China) and quantified.

### 2.4 Cell Counting Kit-8 (CCK-8) assay

Cell viability was assessed using the CCK-8 (Beyotime, China). Absorbance was measured at 450 nm using a microplate reader (Bio-Rad, USA).

### 2.5 Flow cytometry for apoptosis

Apoptosis was assessed using the Annexin V-FITC/PI Apoptosis Detection Kit (Beyotime, China). Samples were analyzed with a flow cytometer (BD, USA), and the data were processed using the FlowJo software.

### 2.6 ROS assay

Intracellular ROS levels were measured with the ROS Assay Kit (Beyotime, China). Fluorescence was detected using a microscope (Olympus, Japan), the images were captured, and fluorescence intensity was quantified using ImageJ.

### 2.7 Enzyme-linked immunosorbent assay (ELISA)

The concentrations of TNF-α and IL-6 in the cell culture supernatants were determined using ELISA kits (Beyotime, China), and the corresponding absorbance was measured at 450 nm using a microplate reader (Bio-Rad, USA).

### 2.8 Statistical analysis

The data are expressed as mean ± SD. Statistical analyses were performed using GraphPad Prism 8 software. One-way ANOVA followed by Tukey’s *post hoc* test was used to determine differences, with a *p*-value of less than 0.05 considered statistically significant.

## 3 Results

### 3.1 SIK1 is downregulated in an *in vitro* model of OA

**Figure 1:**
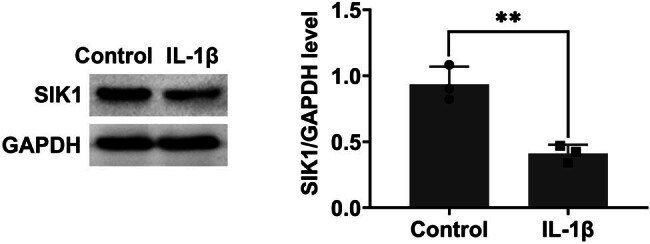
SIK1 is downregulated in an *in vitro* model of osteoarthritis. Immunoblot analysis was performed to assess the expression levels of SIK1 in C28/I2 cells treated with either control or IL-1β for 24 h. Data are presented as mean ± SD. **p < 0.01 versus control.

To investigate the expression of SIK1 in OA, we utilized an *in vitro* model of C28/I2 chondrocytes treated with IL-1β for 24 h. Immunoblot analysis revealed a significant downregulation of SIK1 in the IL-1β-treated group (Figure 1), which indicates that IL-1β treatment negatively affects SIK1 expression in chondrocytes.

### 3.2 Overexpression of SIK1 inhibits IL-1β-stimulated chondrocyte apoptosis

**Figure 2:**
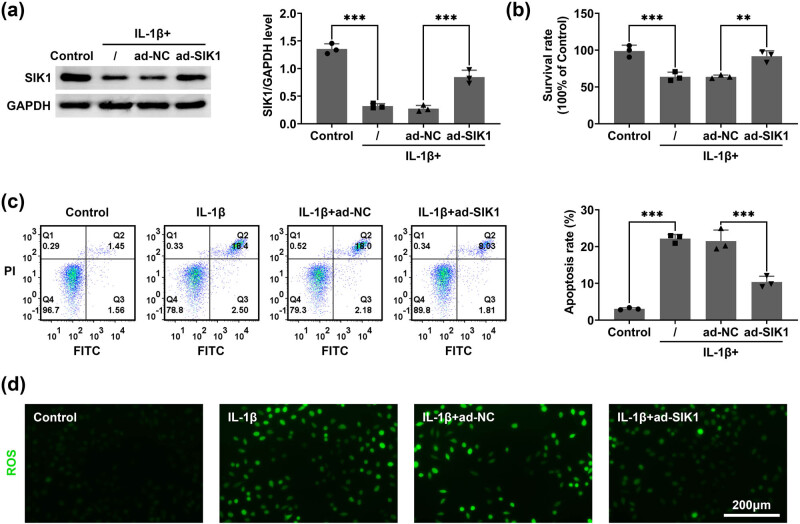
Overexpression of SIK1 inhibits IL-1β-stimulated chondrocyte apoptosis. (a) Immunoblot analysis was performed to assess SIK1 expression levels in C28/I2 cells treated with either control or IL-1β, and infected with ad-NC or ad-SIK1 for 24 h. (b) CCK-8 assay was used to evaluate the impact of SIK1 overexpression on cell viability in C28/I2 cells, following treatment with control or IL-1β and infection with ad-NC or ad-SIK1 for 24 h. (c) Flow cytometry was employed to measure apoptosis rates in C28/I2 cells under the same conditions. (d) ROS assay was conducted to analyze ROS levels in C28/I2 cells, with ROS visualized in the green channel. Scale bar, 200 μm. Data are presented as mean ± SD. **p < 0.01, ***p < 0.001.

Next, we assessed the impact of SIK1 overexpression on IL-1β-induced chondrocyte apoptosis. Immunoblot analysis showed that overexpression of SIK1 via ad-SIK1 infection increased SIK1 protein levels in C28/I2 cells under IL-1β treatment for 24 h (Figure 2a). The CCK-8 assay demonstrated enhanced cell viability in C28/I2 cells with SIK1 overexpression compared to the ad-NC group (Figure 2b). Moreover, flow cytometry analysis revealed a reduction in the apoptosis rate of C28/I2 cells overexpressing SIK1 under IL-1β treatment (Figure 2c). Additionally, the ROS assay showed that SIK1 overexpression decreased ROS levels in C28/I2 cells subjected to IL-1β treatment (Figure 2d). These findings collectively indicate that SIK1 overexpression mitigates IL-1β-induced apoptosis and oxidative stress in chondrocytes.

### 3.3 Overexpression of SIK1 inhibits IL-1β-stimulated chondrocyte inflammation

**Figure 3:**
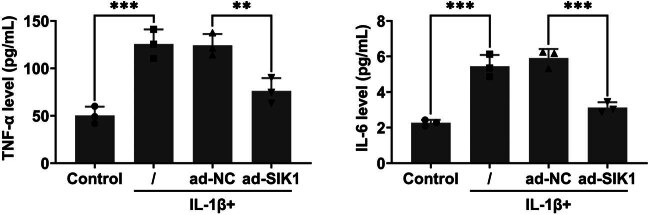
Overexpression of SIK1 inhibits IL-1β-stimulated chondrocyte inflammation. ELISA assays were performed to measure the secretion of TNF-α (left) and IL-6 (right) from C28/I2 cells treated with control or IL-1β and infected with either ad-NC or ad-SIK1 for 24 h. Data are presented as mean ± SD. **p < 0.01, ***p < 0.001.

We further evaluated the effect of SIK1 overexpression on the inflammatory response in IL-1β-stimulated chondrocytes. ELISA assays revealed that SIK1 overexpression reduced the secretion of TNF-α and IL-6 in C28/I2 cells treated with IL-1β, compared to control and ad-NC groups (Figure 3). These results suggest that SIK1 overexpression effectively suppresses the inflammatory response triggered by IL-1β in chondrocytes.

### 3.4 Overexpression of SIK1 promotes the activation of the CRTC2/CREB1 signaling pathway

**Figure 4:**
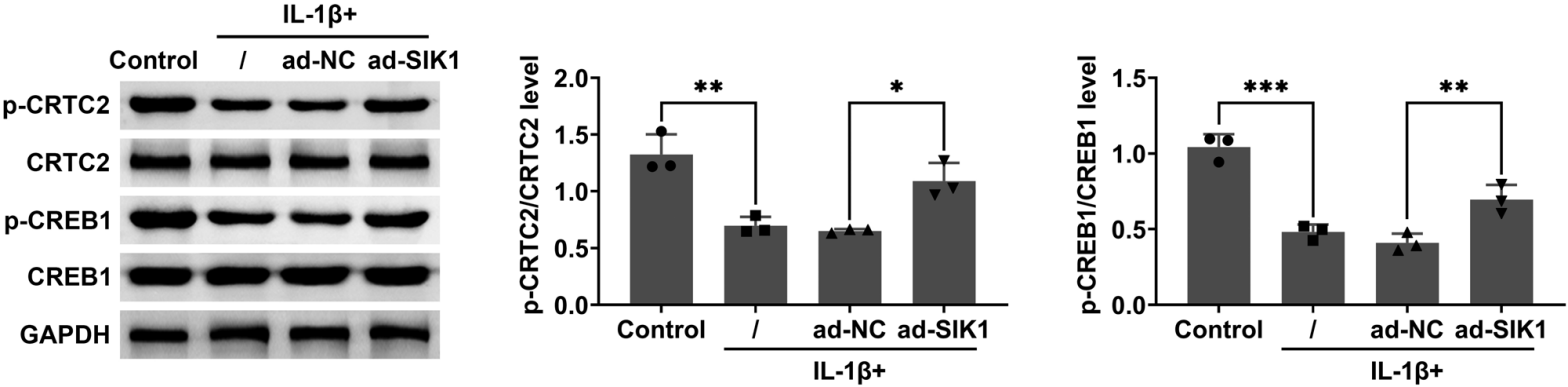
Overexpression of SIK1 promotes the activation of the CRTC2/CREB1 signaling pathway. Immunoblot analysis was conducted to evaluate the expression and phosphorylation levels of CRTC2 and CREB1 in C28/I2 cells treated with control or IL-1β for 24 h. Data are presented as mean ± SD. *p < 0.05, **p < 0.01.

To elucidate the mechanisms underlying the effects of SIK1, we investigated the activation of the CRTC2/CREB1 signaling pathway in IL-1β-treated chondrocytes. Immunoblot analysis was performed to assess both the expression and phosphorylation levels of CRTC2 and CREB1 in C28/I2 cells subjected to IL-1β treatment. Our results indicated that overexpression of SIK1 led to increased phosphorylation of both CRTC2 and CREB1. This finding was corroborated by densitometric analysis, which confirmed elevated phosphorylation levels of CRTC2 and CREB1 (Figure 4). These observations collectively suggest that SIK1 overexpression activates the CRTC2/CREB1 signaling pathway in chondrocytes exposed to IL-1β.

## 4 Discussion

OA is a prevalent degenerative joint disease that significantly impairs mobility, especially in the elderly [3]. Current treatment modalities primarily offer symptomatic relief, failing to effectively arrest disease progression [16,17]. Thus, elucidating the underlying mechanisms of OA and identifying novel therapeutic targets is essential for the development of more effective treatments. Recent research has highlighted the potential role of various molecular pathways in OA, among which SIK1 has emerged as a promising candidate. In this present study, we investigated the role of SIK1 in OA, focusing on its impact on chondrocyte apoptosis and inflammation.

Apoptosis and inflammation are critical processes in OA pathogenesis [18,19]. Chondrocyte apoptosis contributes to cartilage degradation, a defining feature of OA, while inflammation exacerbates joint damage and pain. This study found that SIK1 is downregulated in an *in vitro* model of OA. Notably, the overexpression of SIK1 significantly inhibited IL-1β-stimulated chondrocyte apoptosis and inflammation. These findings indicate that SIK1 plays a protective role in maintaining cartilage integrity and suggest its potential as a therapeutic target for OA.

SIK1, a serine/threonine kinase, is known for its regulatory roles in various physiological processes, including metabolism and circadian rhythms [9]. In the context of OA, our results demonstrate that SIK1 overexpression can mitigate IL-1β-induced apoptotic and inflammatory responses in chondrocytes, which implies that SIK1 may help preserve cartilage by preventing cell death and reducing inflammatory mediators. The protective effects of SIK1 on chondrocytes underscore its potential utility in developing new treatments aimed at preserving joint function in OA patients.

Furthermore, SIK1’s role extends beyond OA to several other diseases. It has been shown to regulate gluconeogenesis in diabetes, modulate cell growth in breast cancer through the p53 and mTOR pathways, and influence bone metabolism [14,20]. These diverse functions of SIK1 illustrate its context-dependent actions, which vary based on the specific cellular environment. In OA, our findings suggest that SIK1’s ability to inhibit apoptosis and inflammation could be harnessed to create targeted therapies addressing the complex nature of the disease.

CRTC2 and CREB1 are pivotal proteins involved in regulating metabolism and stress responses [21]. The CRTC2/CREB1 signaling axis is essential for mediating inflammatory and apoptotic responses. Specifically, CRTC2, a co-activator of the cyclic AMP-response element binding protein (CREB), plays a significant role in maintaining glucose homeostasis in the liver and in several inflammatory diseases [21]. This study found that SIK1 overexpression enhances the activation of the CRTC2/CREB1 pathway in chondrocytes. This activation likely represents a key mechanism through which SIK1 exerts its protective effects against IL-1β-induced apoptosis and inflammation. By promoting the CRTC2/CREB1 signaling, SIK1 may counteract the harmful effects of inflammatory cytokines in OA.

The involvement of the CRTC2/CREB1 pathway in OA is gaining recognition. This pathway influences the expression of genes associated with cell survival and inflammation, thereby playing a crucial role in how chondrocytes respond to inflammatory stimuli. Our findings further support the significance of this pathway in OA, demonstrating that its activation by SIK1 can mitigate IL-1β-induced damage in chondrocytes. These results support the potential of targeting the CRTC2/CREB1 pathway as a therapeutic strategy for OA.

Despite the promising results, this study has some limitations that should be highlighted. The *in vitro* model used does not fully replicate the complex *in vivo* environment of OA-affected joints. To validate our findings, further studies utilizing animal models and clinical samples are necessary. Additionally, the specific molecular interactions between SIK1 and the CRTC2/CREB1 pathway require further investigation. Future research should also address potential side effects and optimize delivery methods for SIK1-based therapies.

In conclusion, this study provides novel insights into the role of SIK1 in OA, illustrating its capacity to inhibit apoptosis and inflammation in chondrocytes through the CRTC2/CREB1 axis. These findings suggest that SIK1 could be a valuable target for developing new OA treatments. Thus, the continued exploration of SIK1’s mechanisms and its interactions with other molecular pathways will be essential for advancing OA therapy and enhancing patient outcomes.
